# Advances and Challenges in Scoring Functions for RNA–Protein Complex Structure Prediction

**DOI:** 10.3390/biom14101245

**Published:** 2024-10-01

**Authors:** Chengwei Zeng, Chen Zhuo, Jiaming Gao, Haoquan Liu, Yunjie Zhao

**Affiliations:** Institute of Biophysics and Department of Physics, Central China Normal University, Wuhan 430079, China; cwzengwuhan@mails.ccnu.edu.cn (C.Z.); chenzhuowh@mails.ccnu.edu.cn (C.Z.); jmgao@mails.ccnu.edu.cn (J.G.); liuhaoquan@mails.ccnu.edu.cn (H.L.)

**Keywords:** RNA-protein complex, scoring function, machine learning, structure prediction, molecular docking

## Abstract

RNA–protein complexes play a crucial role in cellular functions, providing insights into cellular mechanisms and potential therapeutic targets. However, experimental determination of these complex structures is often time-consuming and resource-intensive, and it rarely yields high-resolution data. Many computational approaches have been developed to predict RNA–protein complex structures in recent years. Despite these advances, achieving accurate and high-resolution predictions remains a formidable challenge, primarily due to the limitations inherent in current RNA–protein scoring functions. These scoring functions are critical tools for evaluating and interpreting RNA–protein interactions. This review comprehensively explores the latest advancements in scoring functions for RNA–protein docking, delving into the fundamental principles underlying various approaches, including coarse-grained knowledge-based, all-atom knowledge-based, and machine-learning-based methods. We critically evaluate the strengths and limitations of existing scoring functions, providing a detailed performance assessment. Considering the significant progress demonstrated by machine learning techniques, we discuss emerging trends and propose future research directions to enhance the accuracy and efficiency of scoring functions in RNA–protein complex prediction. We aim to inspire the development of more sophisticated and reliable computational tools in this rapidly evolving field.

## 1. Introduction

RNA–protein complexes are vital for cellular functions, such as DNA repair, RNA splicing, and protein synthesis [1,2,3]. They play a crucial role in gene regulation and the maintenance of chromosome ends [4,5]. Disruptions in RNA–protein interactions are linked to various human diseases, including cancer [6], AIDS [7], and neurodegenerative disorders [8,9]. For example, HIV, a global retrovirus that attacks the human immune system, had caused approximately 39 million infections and 630,000 AIDS-related deaths by the end of 2022. The core mechanism of HIV infection involves an RNA–protein complex, in which the viral protein Tat takes over the host’s positive transcription elongation factor b (P-TEFb) along with the cis-acting transactivation response element (TAR) RNA to regulate transcription elongation. It is crucial to understand the structure of these RNA–protein complexes [7,10]. However, the lack of crystal structures is a major obstacle to developing effective therapies. Therefore, understanding these complexes is crucial for cell biology and for developing targeted therapies [11,12,13].

Understanding RNA–protein interactions requires 3D structural information. However, experimental methods like X-ray crystallography, NMR, and cryo-electron microscopy are costly and time-consuming [14,15]. The high flexibility and complex interaction patterns of RNA make it challenging to determine their structures through experimental methods [16,17]. For instance, it is difficult to achieve high-resolution crystal structures via X-ray crystallography [18], maintain RNA stability during cryo-electron microscopy [19], and obtain precise NMR spectroscopy data for larger RNA molecules [20]. As of 17 August 2024, the Protein Data Bank (PDB) contained 223,790 structures, but only 4888 were RNA–protein complexes [21]. When redundancies were removed, fewer than 400 high-resolution, unique, non-redundant RNA–protein complexes were available for analysis [22,23]. This number is significantly lower than the expected number of RNA–protein complex structures formed within cells.

Therefore, computational structure prediction has gained attention as a viable alternative. A platform for assessing advancements in computational structure prediction is the biennial Critical Assessment of protein Structure Prediction (CASP) competition [24], complemented by CAPRI for protein complexes [25] and RNA-Puzzles for RNA structures [26]. In recent years, CASP challenges have expanded beyond protein structure prediction to include RNA and RNA–protein complex prediction. CASP15 featured two RNA–protein complex targets, and CASP16 introduced additional targets, emphasizing the growing focus on accurately modeling these complex biological interactions. Reliable computational methods can bridge the gap between the scarcity of known RNA–protein structures and the biological processes they control. The demand for accurate theoretical methods to predict the structure of RNA–protein complexes is becoming increasingly urgent. From a computational perspective, RNA–protein complex structure prediction primarily relies on docking, which involves two main steps: conformational sampling and evaluation [27,28,29,30]. The flexibility of RNA and proteins leads to conformational changes, making it difficult to sample near-native structures adequately [31,32]. Sometimes, the sampling process may generate tens of thousands of possible structures, yet none closely resembling the native state [27,33]. Another main challenge lies in conformational evaluation, where a scoring function is used to rank and identify near-native structures among tens of thousands of possible structures [22]. This process is particularly challenging because it requires accurately distinguishing models between near-native structures and others. Existing scoring functions for RNA–protein complexes are based on various assumptions and constructed using different methodologies, each with strengths and limitations.

Several scoring functions have been developed for evaluating the structure of RNA–protein complexes, building on earlier advances in protein and RNA structure prediction [12,22,31]. The cornerstone of this field is knowledge-based scoring functions, which evaluate RNA–protein interactions as a weighted sum of pairwise statistical potentials [34,35]. These statistical potentials utilize formulas derived from the inverse Boltzmann principle. Knowledge-based methods can be categorized into coarse-grained and all-atom approaches. Coarse-grained potentials, such as DARS- RNP, QUASI-RNP [36], and 3dRPC-Score [37], rely on a statistical analysis of interface propensities between nucleotide–residue pairs, capturing pairwise interactions effectively. The coarse-grained representation allows for quicker computational speeds, making them suitable for high-throughput analysis, especially effective when complex formation induces minor structural alterations. In contrast, all-atom potentials, such as dRNA [12], ITScore-PR [22], and DITScore-PR [31], provide higher accuracy when dealing with near-native models among decoys due to their higher spatial resolution. These methods are particularly effective in bound–bound cases where fine structural details are crucial. However, they are less effective in handling unbound–unbound predictions as significant conformational changes occur upon binding.

Deep neural networks have shown potential in recent years across diverse fields, including biophysics in structure prediction [38,39,40]. Well-established methods are available for modeling the 3D structures of proteins [41,42,43,44,45,46], RNAs [47,48,49,50,51,52,53,54], and protein–protein complexes [55,56,57]. Recent studies have shown that machine learning is now excelling in evaluating RNA–protein complex structures. DRPScore, a 4D-CNN-based scoring function, has achieved success comparable to knowledge-based methods in bound–bound cases and has surpassed them in more challenging unbound–unbound cases [23]. These advancements highlight the potential of deep neural networks in revolutionizing the evaluation of RNA–protein complex structures.

**Figure 1:**
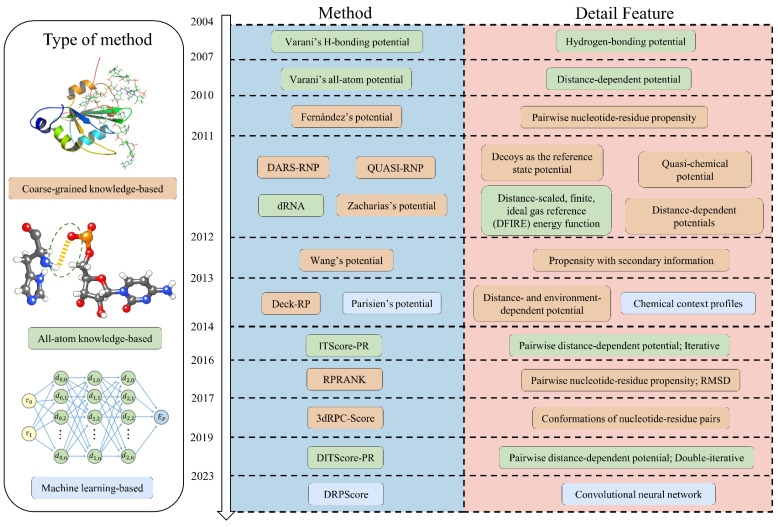
Timeline of the development of RNA–protein complex structure prediction. The most recent advancements in RNA–protein structure prediction encompass coarse-grained knowledge-based, all-atom knowledge-based, and machine-learning-based approaches.

This review provides a comprehensive overview of scoring functions for RNA–protein structure prediction, presenting the latest advances in the field. We discuss the fundamental principles of various scoring functions, including knowledge-based approaches (coarse-grained and all-atom models) and machine-learning-based methods (Figure 1). Moreover, we assess the strengths and limitations of current scoring techniques, offering a detailed evaluation of their performance. Given the significant progress demonstrated by machine learning approaches, we propose future research directions that could further enhance the accuracy and efficiency of scoring functions. We aim to inspire the development of more robust and sophisticated scoring techniques to advance RNA–protein complex structure prediction.

## 2. Knowledge-Based Scoring Functions

**Figure 2:**
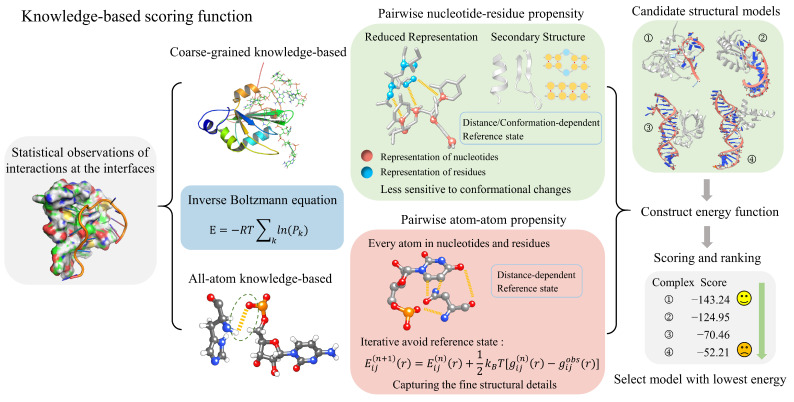
The process and principles of knowledge-based scoring functions in evaluating RNA–protein complexes. These scoring functions can be categorized into coarse-grained and all-atom models derived from the inverse Boltzmann equation. Coarse-grained scoring functions utilize a simplified representation, while all-atom scoring functions account for every atom within nucleotides and residues. Once the energy function is constructed, these scoring functions can evaluate and rank RNA–protein complexes, allowing the selection of structures with the lowest energy scores.

Knowledge-based scoring functions are mathematical functions derived from statistical observations of interactions at the interfaces of known RNA–protein complexes. Figure 2 shows that these functions commonly use the inverse Boltzmann relationship to convert distance-dependent pairwise contact probability distributions into statistical potential functions. This means that specific nucleotide and residue interactions are observed at the RNA–protein interface with a higher frequency than would be expected by random chance [58,59,60].

For instance, the highly negative charge of RNA attracts positively charged amino acids, such as arginine (ARG), lysine (LYS), and histidine (HIS), which are prevalent at the RNA–protein interaction interface [58,61,62]. Electrostatic interactions are crucial for the stabilization of RNA–protein complexes [58,63,64]. Notably, guanine (G) is found at the interface more frequently than cytosine (C), adenine (A), or uracil (U), with its occurrence exceeding 30%. On the other hand, while hydrophobic residues, including aromatic residues, are the least favored at these interfaces, aromatic residues still play an important role in interacting with unpaired RNA bases [65].

Consequently, RNA–protein interactions are primarily mediated by electrostatic forces rather than the hydrophobic forces and desolvation effects that dominate protein–protein binding. It has been observed that the ‘LYS-ARG’ fragment in proteins is a highly favorable binding motif. In RNA, ‘CG’ and ‘GG’ are the preferred binding fragments among the nucleotides [62]. This preference is primarily due to guanine’s unique double-ring structure, which facilitates extensive hydrogen bonding and stacking interactions. These enhance stability and versatility in interactions with amino acids. Knowledge-based scoring functions measure the tendency of these distance-dependent pairwise contacts to occur at the interface [34,35].

The Boltzmann distribution in statistical physics provides a foundation for understanding interaction probabilities in thermal equilibrium. The interaction energy $∆E_{k}$ correlates with the probability distribution $P_{k}$ as follows:

(1) $$P_{k}\propto e^{-\frac{{∆E}_{k}}{RT}}$$

where $P_{k}$ is the probability of the system being in a state *k* where the interaction or distance occurs; the state can either be a specific nucleotide/residue or a specific nucleotide–residue pair. ${∆E}_{k}$ is the interaction energy between states k, *R* is the gas constant, and *T* is the absolute temperature. In previous work, the value of *RT* was set at 0.59 kcal/mol.

Therefore, the inverse Boltzmann distribution can allow us to derive the interaction potential energy. By statistically analyzing the interaction frequencies from existing structural databases, corresponding potential energy functions can be inferred. For example, consider the distribution probability $P_{k}$ of state *k* at the interface. According to the inverse Boltzmann principle, this observed probability distribution can be directly related to the interaction potential energy ${∆E}_{k}$:

(2) $$∆E_{k}=-RTln(P_{k})$$

Therefore, negative statistical potential values indicate favorable binding energies. Thus, the total score ∆E for a given RNA–protein complex is obtained by summing the interaction energies for all specific states at the interface:

(3) $$∆E=\sum_{k}∆E_{k}$$

The next and most crucial step is to analyze the geometric information of interaction pairs at the interaction interfaces of RNA–protein complexes in known structural databases (such as RCSB PDB [21] and NDB [66,67]) to obtain the probability distribution $P_{k}$. In general, for each selected state *k*, the observed frequency $N_{k}$ in all RNA–protein complexes is counted and then normalized to obtain the probability distribution $P_{k}$:

(4) $$P_{k}=\frac{N_{k}}{\sum_{k}N_{k}}$$

where $\sum_{k}N_{k}$ is the total frequency observed across all states.

The definition of state k can differ among research groups. This state may be defined by a general distance range, such as 1 to 5 Å, or by specific criteria tailored to the type of interaction and the involved atomic groups. Several classical formulations exist for constructing the propensity distribution $P_{k}$. As illustrated in Equation (5), the propensity for a nucleotide or a residue of type *k* is classically defined as the ratio of the observed frequencies:

(5) $$P_{k}=\frac{N_{k}^{I}/\sum_{K}N_{k}^{I}}{N_{k}^{A}/\sum_{K}N_{k}^{A}}$$

where $N_{k}^{I}$ is the number of nucleotides/residues of type *k* involved in the interface, and $\sum_{K}N_{k}^{I}$ is the total number of interface nucleotides/residues. $N_{k}^{A}$ is the total number of nucleotides/residues of type *k*, and $\sum_{K}N_{k}^{A}$ is the total number of nucleotides/residues.

The Fernández-Recio group adopted a different propensity definition, emphasizing the surface nucleotides and residues [68]. They defined propensity as

(6) $$P_{k}=\frac{N_{k}^{I}/\sum_{K}N_{k}^{I}}{N_{k}^{S}/\sum_{K}N_{k}^{S}}$$

where $N_{k}^{I}$ and $\sum_{K}N_{k}^{I}$ have the same definitions given in equation (5), $N_{k}^{I}$ is the number of nucleotides/residues of type *k* involved in the interface, and $\sum_{K}N_{k}^{I}$ is the total number of interface nucleotides/residues.$\;N_{k}^{S}$ is the total number of nucleotides/residues of type *k* on the surface, and $\sum_{K}N_{k}^{S}$ is the total number of nucleotides/residues on the surface.

Additionally, the propensity of nucleotide–residue pairs on the interfaces of RNA–protein complexes is used to develop propensity-based statistical potentials. The nucleotide–residue pairs are defined based on a cutoff distance between the nearest atoms [63]. Several statistical potentials based on propensity have been developed for evaluating RNA–protein complexes. These models consider factors such as secondary structure details, relative distances, and orientations between nucleotide–residue pairs. In the upcoming sections, we will provide a detailed analysis emphasizing the selection of state *k* at either the residue level (coarse-grained) or the atomic level (all-atom). By taking this approach, we aim to assess the impact of these different resolutions on the accuracy and applicability of the statistical potentials used in modeling RNA–protein interactions.

### 2.1. Coarse-Grained Knowledge-Based Scoring Functions

Over the last decade, significant progress has been made in developing coarse-grained potentials for evaluating the structure of RNA–protein complexes (see Table 1). Starting with Fernández’s work, which established the basis for distance-dependent pairwise nucleotide–residue scoring [63], subsequent models like DARS-RNP and QUASI-RNP improved on this approach by including more advanced reference states and better representations [36]. The Xiao group further developed this concept with Deck-RP [69], RPRANK [70], and 3dRPC-Score [37], incorporating secondary structure information and utilizing new statistical methods beyond simple distance dependence. These enhancements have broadened the usefulness of coarse-grained potentials, making them more effective in a broader range of structural evaluation situations. The following sections will provide a detailed overview of these methods.

*Fernández’s potential:* In 2010, the Fernández group developed a distance-dependent pairwise nucleotide–residue propensity to score the RNA–protein complexes [63]. In this approach, we can determine the propensities by comparing the observed frequencies of specific nucleotide–residue pairs (where *i* = 1 to 4 for nucleotides and *j* = 1 to 20 for residues) at the RNA–protein interface with their expected frequencies.

(7) $$P_{ij}=\frac{N_{ij}^{I}/\sum_{ij}N_{ij}^{I}}{N_{i}^{S}/\sum_{i}N_{i}^{S}\times N_{j}^{S}/\sum_{j}N_{j}^{S}}$$

where $N_{ij}^{I}$ is the number of pairs between nucleotide type *i* and residue type *j* at the interface, $\sum_{ij}N_{ij}^{I}$ is the total number of nucleotide–residue pairs at the interface, and $N_{i}^{S}$ and $N_{j}^{S}$ are the number of nucleotides of type *i* and the number of residues of type *j* on the interface, respectively, while $\sum_{i}N_{i}^{S}$ and $\sum_{j}N_{j}^{S}$ are the total number of nucleotides and residues on the surface, respectively. These expected frequencies are based on the overall composition of RNA and protein surfaces. The nucleotide–residue pairs were defined by having at least one atom within a cutoff distance of 4 Å from each other, which serves as the distance threshold for defining a contact. Additionally, the surface nucleotide or residue was defined as that with an ASA (accessible surface area) > 0.1 Å^2^. This distance-dependent potential uses a cutoff distance to evaluate contacts between nucleotide–residue pairs. While this approach is less sensitive to subtle differences in model structures, particularly those that share identical contact pairs, it offers the advantage of being more resilient to minor conformational changes. This tolerance makes it useful in scenarios where slight structural variations are expected. Still, this approach cannot fully capture the highly detailed interactions within the complex model. Unfortunately, this potential was designed to improve the discriminative power of the FTDock potential and is not available as a standalone program.

*DARS-RNP and QUASI-RNP:* In 2011, Tuszynska and Bujnicki introduced two medium-resolution, coarse-grained potentials—namely, the quasi-chemical potential (QUASI-RNP) and the Decoys As the Reference State potential (DARS-RNP)—to evaluate RNA–protein complex structures [36]. This coarse-grained methodology simplifies the all-atom representation of macromolecular structures into a reduced form based on nucleotide or residue type [73]. The backbone is represented by two united atoms for nucleotides: one for the phosphate group (P) and one for the ribose (RIB). Pyrimidines are modeled with a single atom, while purines are represented with two atoms. Conversely, residues are depicted using one to three united atoms, depending on their molecular size.

These two potentials, QUASI-RNP and DARS-RNP, use the same mathematical base but differ in their reference state:

(8) $$E=E_{d}+E_{a}+E_{s}+E_{p}$$

where *E* is the total energy term, and $E_{d}$, $E_{a}$, $E_{s}$, and $E_{p}$ are the distance-dependent, angular-dependent, site-dependent, and penalty terms for steric clashes, respectively. All four terms of the energy function are equally weighted for they exhibit comparable values. Among these energy terms, the interaction energies $E_{d}$, $E_{a}$, and $E_{s}$ between the united atom type from the RNA *i* and the united atom type from the protein *j* are calculated by the same formula:

(9) $$E(i,j,d)=-RTln\frac{N_{obs}(i,j,d)}{N_{exp}(i,j,d)}$$

where *E* represents $E_{d}$, $E_{a},$ or $E_{s}$,$\;N_{obs}(i,j,d)$ denotes the number of observed contacts between atom types i and *j* within a specific distance or angular bin d in the training set, and $N_{exp}(i,j,d)$ refers to the expected number of contacts within the same distance/angular bin in the reference state. The bin size is not standardized and is determined through empirical testing. There are two bin types: a distance bin of 1 Å used for the distance-dependent term $E_{d}$, and an angular bin of 20° used for the angular-dependent term $E_{a}$. The energy for each RNA–protein united atom pair is calculated when they are within 9 Å of each other. For the site-dependent term $E_{s}$, the parameter *d* represents one of three interaction types between residues and nucleotide edges: Watson–Crick, Sugar, or Hoogsteen edges [74]. The penalty term $E_{p}$ for steric clashes restricts united atom pairs, preventing them from approaching within a predefined cutoff distance.

It is essential to address the reference state problem when creating a distance-dependent statistical potential energy between pairs of particles. While calculating $N_{obs}(i,j,d)$, from a given training dataset, the observed number of contacts between atom types *i* and *j* with bin d is straightforward. Still, estimating the expected contact number, $N_{exp}(i,j,d)$, is more challenging. The expected distribution of paired nucleotide residues over distances can be adjusted using valid reference state definitions, including mean reference states, quasi-chemical approximate reference states, and finite ideal-gas reference states. QUASI-RNP and DARS-RNP employ distinct methods to determine the reference state in their calculations. For QUASI-RNP, molar fractions of residues are used to calculate $N_{exp}(i,j,d)$:

(10) $$N_{exp}\left(i,j,d\right)=X_{i}*X_{j}*N_{obs}(d)$$

where $X_{i}$ and $X_{j}$ are the molar fractions of atom types *i* and *j* in the given training set, respectively, and $N_{obs}(d)$ is the total number of contacts in bin *d* irrespective of atom type. For DARS-RNP, $N_{exp}\left(i,j,d\right)$ is a normalized number of contacts between atom types *i* and *j* in bin *d*, calculated from 1000 decoys generated by the docking program GRAMM [75] for each RNA–protein complex in the training set. In both bound and unbound docking tests, DARS-RNP demonstrated a slightly stronger performance than QUASI-RNP to identify near-native structures. DARS-RNP is constructed from a much more extensive training set, providing a more realistic representation of “random” protein–RNA interactions. These two scoring functions are designed to be less affected by structural changes. As a result, they are expected to be more effective in distinguishing between different structures when complex formation causes only small changes. Furthermore, these functions provide a better spatial resolution and a more accurate representation of the reference state, leading to an improved accuracy in distinguishing between near-native structures and decoys compared to Fernandez’s potential. In cases where molecules are already bound together, these functions were able to effectively differentiate between similar RNA–protein complex structures with small differences (RMSD < 10 Å). These functions showed a competitive discriminative ability in more challenging cases where the molecules were not initially bound together. The package of the model is freely available at https://genesilico.pl/software/stand-alone/statistical-potentials (accessed on 29 September 2024).

*Zacharias’s potential:* In 2011, The Zacharias group developed a distance-dependent, coarse-grained force field for RNA–protein docking [71]. This potential enables fully systematic docking through energy minimization in the binding partners’ rotational and translational degrees of freedom. In this coarse-grained approach, each residue is represented by up to four pseudo atoms (beads): two for the main chain nitrogen (N) and oxygen (O), and one or two for the short and long side chains, respectively. For nucleotides, three pseudo atoms represent the phosphate/ribose part, and three or four represent purine and pyrimidine bases. There are 31 pseudo-atom types for proteins and 17 pseudo-atom types for RNA.

This potential assumed pairwise additive interactions between protein and RNA beads, which are described by a distance-dependent potential with two forms, corresponding to attractive and repulsive interactions. The attractive potential is of the Lennard-Jones type:

(11) $$U_{ij}^{attr}=\epsilon_{ij}(\frac{\sigma_{ij}}{r^{8}}-\frac{\sigma_{ij}}{r^{6}})$$

The repulsive potential is

(12) $$U_{ij}^{rep}(r)=\left\{\begin{array}{c} U_{ij}^{attr}\left(r\right)+2U_{ij}^{m},\;\;\;\;r\leq r_{ij}^{m}\\ -U_{ij}^{attr}\left(r\right),\;\;\;\;\;\;\;\;\;r>r_{ij}^{m} \end{array}\right.$$

Two pairwise-specific parameters $\sigma_{ij}$ and $\epsilon_{ij}$ describe the interaction of each pair *ij* of RNA and protein beads, governing the interaction range and strength, respectively. $r_{ij}^{m}$ and $U_{ij}^{m}$ correspond to the position and minimum value of $U_{ij}^{attr}$. Thus, there are, in total, 1054 parameters (31 × 17 × 2) that need to be derived in a knowledge-based manner. Similar to DARS-RNP and QUASI-RNP [36], the distance-dependent statistical potentials E(i,j,d) were constructed for each bead pair by a set of RNA–protein complexes determined through Equation (9). Then, the initial values of σ and ϵ parameters were obtained by fitting the attractive potential in Equation (11) and repulsive potential in Equation (12) to E(i,j,d). The parameter values were subsequently adjusted to optimize docking results, aiming to find the correct (close to native) binding mode and achieve appropriate scoring. These potentials can accommodate moderate conformational changes but cannot be used without the ATTRACT docking protocol.

*Wang’s potential:* In 2012, the Wang group developed four pairwise nucleotide–residue propensity potentials from a given training set, depending on whether the secondary structure element (SSE) information of RNA and proteins was considered [72]. Based on the propensity values of protein SSEs, eight types of SSEs calculated by the DSSP program were categorized into three classes: X (π-helix “I”, $3_{10}$-helix “G”, and bend “S”, whose *p* > 1), Y (β-sheet “E”, β-bridge “B”, turn “T”, and unclassified, whose *p* ≈ 1), and Z (α-helix “H”, whose *p* < 1). Similarly, three types of nucleotides calculated by the X3DNA program were categorized into two classes: NP (unpaired and non-WC paired nucleotides, whose *p* > 1) and P (WC paired nucleotides, whose *p* < 1). Therefore, similar to Equation (9), the propensity can be calculated from the observed probability of the specific residue–nucleotide pair of type ai−bj (where *a* = 1…20 for residues, *i* = X, Y, Z for protein secondary structure classes, *b* = 1…4 for nucleotides, *j* = P, NP for RNA secondary structure classes) at interfaces, divided by the expected probability. The authors concluded that the RNA secondary structure information plays a more significant role than the protein secondary structure in accurately discriminating the RNA–protein complex structures. Unfortunately, this potential is not available as a standalone program.

*Deck-RP:* In 2013, the Xiao group developed Deck-RP, a distance- and environment-dependent potential specifically designed for RNA–protein complexes generated by RPDOCK [69]. Deck-RP merges the strengths of both Wang’s potential and DARS-RNP by incorporating an enhanced reference state that accounts for propensities, secondary structure states, and interface preferences of nucleotides and residues. The reference state in Deck-RP is a hybrid, composed of a decoy-based component and a molar-fraction-corrected component. The decoy-based component takes account of all decoys in the training set as the reference state, while the molar-fraction-corrected component takes account of the interface concentration or specific preferences of nucleotides and residues. As a result, similar to Equation (9), the propensity of residue–nucleotide pairs can be derived from their observed probabilities. The model considers 168 unique nucleotide–residue pairs, encompassing four nucleotide types across two secondary structure states and seven residue types across three secondary structure states. The 3dRPC protocol includes the docking program RPDOCK and the scoring program Deck-RP, which has been developed into a user-friendly webserver version at http://biophy.hust.edu.cn/new/3dRPC (accessed on 29 September 2024), while the package is freely available at http://biophy.hust.edu.cn/new/resources/3dRPC (accessed on 29 September 2024).

*RPRANK:* In 2016, the Xiao group developed a new knowledge-based potential, RPRANK, using root mean square deviation (RMSD) as a measure [70]. Unlike the previous statistical potential, RPRANK does not use distance to classify the residue-base pairs directly. The conformational differences between nucleotide–residue pairs from decoys and standard pairs from native structures were used to calculate the statistical potential. The nucleotide–residue pairs are clustered based on the RMSD between each other. Then, the energies of the nucleotide–residue pair clusters are decided by a statistical method based on the number of pairs in each cluster. The 3dRPC protocol includes the docking program RPDOCK and the scoring program RPRANK, which has been developed into a user-friendly webserver version at http://biophy.hust.edu.cn/new/3dRPC (accessed on 29 September 2024). The package is freely available at http://biophy.hust.edu.cn/new/resources/3dRPC (accessed on 29 September 2024).

*3dRPC-Score:* In 2017, the Xiao group introduced a new statistical potential called 3dRPC-Score [37]. Unlike the commonly used distance-dependent statistical potential, this method considers the conformations of nucleotide–residue pairs as statistical variables. The group proposed that accurately defining the energy of a nucleotide–residue pair requires considering not only the relative distance between the partners but also their relative distance and orientation. They classified the nucleotide–residue pairs into 10 classes based on the relative root mean square deviation (RMSD) between their conformations. This classification allows pairs with similar conformations to be considered to have the same energy. Therefore, the statistical potential $E_{ij}(C)$ could be calculated:

(13) $$E_{ij}\left(C\right)=-ln(\frac{P_{ij}(C)}{P_{i}P_{j}*P_{v}})$$

where $P_{ij}(C)$ is the occurrence probability of the pair of i-type nucleotide and *j*-type residue in class *C*, $P_{i}$ and $P_{j}$ are the probabilities of nucleotide *i* and residue *j* at the interface, respectively, and $P_{v}$ is the probability of class *C* in the whole conformational space of nucleotide–residue pairs in an ideal state. In an ideal state, each class of nucleotide–residue pairs has the same probability in conformational space. Thus,

(14) $$E_{ij}\left(C\right)=-\ln\left(\frac{P_{ij}\left(C\right)}{P_{i}P_{j}}\right)+constant$$

where the constant = $lnP_{v}$. The scoring function performs best when the constant is set as −4. The 3dRPC webserver is available at http://biophy.hust.edu.cn/new/3dRPC (accessed on 29 September 2024). The package can be downloaded at http://biophy.hust.edu.cn/new/resources/3dRPC (accessed on 29 September 2024).

Coarse-grained representations are less sensitive to conformational changes, which makes them suitable for high-throughput scenarios and situations involving minor to moderate conformational changes. These representations are expected to have greater discriminatory power when complex formation induces only minor structural alterations in its components. However, they may struggle to capture fine structural details and complex molecular interactions, especially when dealing with significant conformational changes. In such cases, these methods may need to be supplemented with higher-resolution techniques for tasks requiring detailed structural analysis or when addressing more complex challenges.

### 2.2. All-Atom Knowledge-Based Scoring Functions

The development of all-atom knowledge-based scoring functions for evaluating RNA–protein complexes has progressed from basic models to more advanced techniques (Table 2). The Varani group initially created a hydrogen-bonding potential to lay the foundation for specific recognition between proteins and RNA based on the sequence [11]. Later, realizing that hydrogen bonds only represent a fraction of the interactions at RNA–protein interfaces, they introduced an all-atom, distance-dependent potential to improve the accuracy of structural predictions [76]. Building on this work, the Zhou group developed dRNA, using a carefully constructed reference state to enhance the accuracy of pairwise potentials [12]. Subsequently, the Zou and Huang groups advanced the field with ITScore-PR [22] and DITScore-PR [31], both employing iterative processes to improve potentials and effectively eliminate the need for a predefined reference state, thus enhancing accuracy and applicability, especially in the consideration of fine structural details in RNA–protein interactions. In the following sections, we will provide a detailed overview of each of these methods.

*Varani’s H-bonding potential:* In 2004, the Varani group developed an atomic-level, distance- and orientation-dependent hydrogen-bonding (H-bond) potential [11]. This hydrogen-bonding potential consists of a distance-dependent energy term [$E(\delta_{HA})$] and three angular-dependent energy components: *E(Θ)* (the angle at the hydrogen atom), *E(Ψ)* (the angle at the acceptor atom), and *E(X)* (the dihedral angle of the hydrogen bond). The total hydrogen-bond energy ($E_{HB})$ is then derived as a linear combination of these four distance- and orientational-dependent terms under the assumption that they are independent of each other:

(15) $$E_{HB}=E\left(\delta_{HA}\right)+E\left(\Theta \right)+E\left(\Psi \right)+E(X)$$

However, hydrogen bonds represent only approximately 25% of the contacts at RNA–protein complex interfaces. A more comprehensive approach is needed to effectively describe the full types of interactions occurring at these interfaces. Unfortunately, this potential is not available as a standalone program.

*Varani’s all-atom potential:* In 2007, the Varani group developed a distance-dependent statistical potential for predicting sequence-specific recognition between proteins and RNA, building upon the previous H-bonding potential, which represents only one aspect of the complex interactions at play [76]. This all-atom potential treats every atom, in every nucleotide and residue, as a unique type (e.g., Ala $C_{\beta }$ and Arg $C_{\beta }$ are considered unique atom types under this scheme), resulting in a total of 158 protein and 81 RNA atom types. Chemically similar atoms were grouped together based on the CHARMM atom definitions, allowing interactions between these atoms to be treated consistently. This potential is useful for distinguishing between RNA–protein complex models similar to the native structure, particularly those with a root mean square deviation (RMSD) of less than 5 Å. However, in practical unbound–unbound cases, obtaining a significant number of decoys with RMSD < 5 Å is challenging. This potential is not available as a standalone program.

*dRNA:* In 2011, Zhou group developed dRNA, a volume-fraction-corrected, distance-scaled, finite, ideal gas reference (DFIRE) statistical energy function and a measure of relative structural similarity by Z-score for RNA–protein complex interactions [12]. The definition of the reference state is critical for developing distance-dependent potentials accurately. The reference state serves as the basis for comparing observed interactions, and its precise formulation is essential for the reliability of the statistical potential. However, accurately determining the functional form of the reference state remains a significant challenge. Errors in the reference state can result in inaccuracies in the calculated potentials, especially when pairwise interactions are inaccurately represented or abrupt truncations are applied [77,78]. The final statistical energy $E_{ij}(r)$ could be calculated as follows:

(16) $$E_{ij}\left(r\right)=\left\{\begin{array}{c} -\eta ln\frac{N_{obs}\left(i,j,r\right)}{{\left(\frac{f_{i}^{v}\left(r\right)f_{j}^{v}\left(r\right)}{f_{i}^{v}\left(r_{cut}\right)f_{j}^{v}\left(r_{cut}\right)}\right)}^{\beta }\frac{r^{\alpha }∆r}{r_{cut}^{\alpha }∆r_{cut}}N_{obs}^{lc}\left(i,j,r_{cut}\right)},\;r<r_{cut}\\ \;\;\;\;\;\;\;\;\;\;\;\;\;\;\;\;\;\;\;\;\;\;0\;\;\;\;\;\;\;\;\;\;\;\;\;\;\;\;\;\;,\;r\geq r_{cut} \end{array}\right.$$

where the volume-fraction factor $f_{i}^{v}\left(r\right)$ is

(17) $$f_{i}^{v}\left(r\right)=\frac{\sum_{j}N_{obs}^{RNA-protein}(i,j,r)}{\sum_{j}N_{obs}^{All}(i,j,r)}$$

where $N_{obs}\left(i,j,r\right)$ is the number of pairs of atom *i* and atom *j* within the spherical shell at distance r observed in a given RNA–protein complex structure database, and the interaction cutoff distance $r_{cut}$ is 15 Å. $∆r_{cut}$, the bin width at $r_{cut}$, is 0.5 Å. The value of α is set to 1.61, determined by the best fit of $r^{\alpha }$ to the actual distance-dependent number of ideal-gas points in finite protein-sized spheres. The value of β for volume correction is set to 0.5. The factor η is 0.01 to control the magnitude of the energy score. Similar to Varani’s all-atom potential, this all-atom potential also treats every atom, in every nucleotide and residue, as a unique type, resulting in a total of 167 protein and 86 RNA atom types. dRNA offers an improved accuracy in distinguishing low-RMSD, near-native models from thousands of decoys compared to coarse-grained scoring functions, primarily due to its higher spatial resolution inherent in the energy function. Unfortunately, this potential is not available as a standalone program.

*ITScore-PR:* In 2014, the Zou group developed a pairwise distance-dependent atomic interaction potential, ITScore-PR, using a statistical mechanics-based iterative method [22]. ITScore-PR addresses the reference state problem by iteratively improving the interatomic pair potentials. This is achieved by comparing RNA–protein complexes’ predicted pair distribution functions with the experimentally observed pair distribution functions of native crystal structures in a specific training set. The potential $E_{ij}(r)$ over all atom pairs *ij* in the RNA and protein is determined through an iterative formula:

(18) $$E_{ij}^{\left(n+1\right)}\left(r\right)=E_{ij}^{\left(n\right)}\left(r\right)+∆E_{ij}^{n}(r)$$

(19) $$∆E_{ij}^{n}\left(r\right)=\frac{1}{2}k_{B}T\;[g_{ij}^{\left(n\right)}\left(r\right)-g_{ij}^{obs}(r)]$$

where *n* denotes the iterative step, and $E_{ij}^{\left(n+1\right)}\left(r\right)$ are the improved potentials from $E_{ij}^{\left(n\right)}\left(r\right)$ after correction, used in the next iterative step. The separations *r* between atom *i* and atom *j* are divided into bins of 0.2 Å with a maximum cutoff value of 10 Å. $g_{ij}^{\left(n\right)}\left(r\right)$ and $g_{ij}^{obs}(r)$ stand for the pair distribution functions for atom pair *ij*, calculated according to $E_{ij}^{\left(n\right)}\left(r\right)$ and calculated from the native crystal structures in the training set, respectively. $g_{ij}^{obs}\left(r\right)$ is calculated by the following:

(20) $$g_{ij}^{obs}\left(r\right)=\frac{1}{k}\sum_{k=1}^{K}g_{ij}^{k*}(r)$$

where *K* is the total number of the RNA–protein complexes in the training set, and $g_{ij}^{k*}(r)$ is the pair distribution function of the *k*-th native complex structure. $g_{ij}^{\left(n\right)}\left(r\right)$ is the pair distribution function calculated from the ensemble of the binding modes according to the binding score-dependent Boltzmann probabilities $P_{k}^{l}$ obtained from the potential $E_{ij}^{\left(n\right)}\left(r\right)$ at the *n*-th step.

(21) $$g_{ij}^{\left(n\right)}\left(r\right)=\frac{1}{k}\sum_{k=1}^{K}\sum_{l=0}^{L}P_{k}^{l}g_{ij}^{kl}(r)$$

where $g_{ij}^{kl}(r)$ is the pair distribution function for atom pair *ij* observed in the *l*-th binding state of the *k*-th complex. Thus, for a given set of initial potentials $E_{ij}^{\left(0\right)}\left(r\right)$,

(22) $$E_{ij}^{\left(0\right)}\left(r\right)=\left\{\begin{array}{cc} w_{ij}\left(r\right) & ,\;\mathrm{for}\;\mathrm{hydrogen}\;\mathrm{bond}\;\mathrm{pairs}\\ \frac{v_{ij}\left(r\right)e^{-v_{ij}\left(r\right)}+w_{ij}\left(r\right)e^{-w_{ij}\left(r\right)}}{e^{-v_{ij}\left(r\right)}+e^{-w_{ij}\left(r\right)}}\; & ,\;\mathrm{otherwise} \end{array}\right.$$

where $v_{ij}\left(r\right)$ is the van der Waals (VDW) potential by ZDOCK 2.1, and $w_{ij}\left(r\right)=-k_{B}Tlng_{ij}^{obs}(r)$ is the potential of mean force. The iteration continues through Equations (18)–(21) until all native structures in the training set can be discriminated from decoys by the current potentials. 12 RNA atom types and 20 protein atom types are used in this statistical potential. ITScore-PR clearly outperforms other scoring functions using detailed all-atom representation and an iterative processing approach, especially in bound–bound cases. The package is available at https://zoulab.dalton.missouri.edu/resources_itscorepr.html (accessed on 29 September 2024).

*DITScore-PR:* In 2019, building on ITScore-PR [22], the Huang group developed a set of effective pair potentials, DITScore-PR, for protein–RNA interactions using a double-iterative method [31]. This algorithm circumvents the reference state problem by updating the potentials until they can effectively distinguish native structures from binding decoys. It overcomes the decoy-dependent limitation by iteratively constructing the binding decoys. Similar to ITScore-PR but with a distinct approach, DITScore-PR consists of an inner loop and an outer loop for the two iteration processes:

(23) $$E_{ij}^{\left(n,k+1\right)}\left(r\right)=E_{ij}^{\left(n,k\right)}\left(r\right)+\frac{1}{2}k_{B}T\;[g_{ij}^{\left(n,k\right)}\left(r\right)-g_{ij}^{obs}\left(r\right)]$$

where *n* and *k* stand for the iterative indices of the outer and inner loops, *i* and *j* represent the types of protein and RNA atoms, $g_{ij}^{\left(n,k\right)}\left(r\right)$ is the predicted pair distribution function by the current potentials $E_{ij}^{\left(n,k\right)}\left(r\right)$ at the *k*-th inner iteration cycle for a fixed *n*-th iterative cycle, and $g_{ij}^{obs}\left(r\right)$ is the experimentally observed pair distribution function in the native complex structures of a training set. The outer loop conducts one iteration cycle when the inner loop is completed once. The outer loop repeats until the iterative steps reach a set number or the inner loop is converged. The definition for RNA and protein atom types is the same as in ITScore-PR, which gave 12 RNA atom types and 20 protein atom types. The separations r between atom *i* and atom *j* are divided into bins of 0.2 Å with a maximum cutoff value of 9 Å. DITScore-PR demonstrates a higher accuracy than coarse-grained potentials in bound–bound cases, achieving a success rate of approximately 80%. However, while outperforming other approaches in the more challenging unbound–unbound cases, the success rate still requires improvement. In true flexible docking processes, where binding partners can adapt to each other, the performance of DITScore-PR remains limited in handling significant conformational changes. The package of the model is freely available at http://huanglab.phys.hust.edu.cn/mprdock/ (accessed on 29 September 2024).

Overall, all-atom potentials provide a superior accuracy and resolution in capturing the detailed atomic interactions of RNA–protein complexes compared to coarse-grained potentials. These methods are highly effective in bound–bound cases, outperforming coarse-grained potentials by offering more precise discrimination of near-native structures. However, the enhanced resolution of all-atom methods comes with increased computational complexity and a reduced performance in facing complex conformational changes. This limitation is particularly pronounced in true flexible docking scenarios, where binding partners must adapt to substantial conformational changes [79]. Therefore, while all-atom potentials offer significant advantages in high-precision applications, their limitations in flexibility suggest that they may need to be supplemented with other techniques, especially in situations involving substantial conformational changes.

## 3. Machine-Learning-Based Scoring Functions

**Figure 3:**
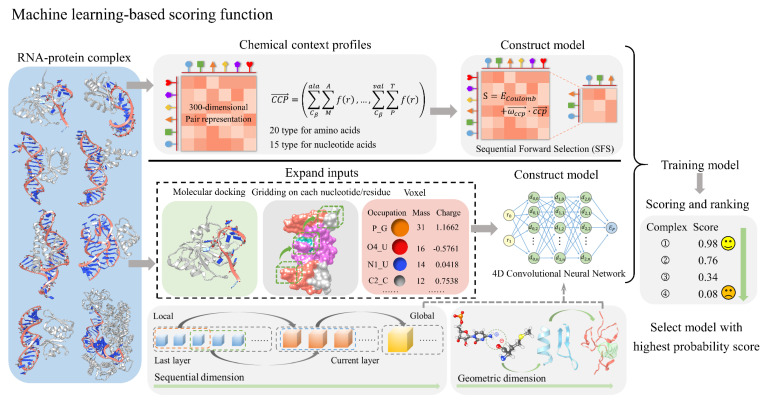
The process and principles of machine-learning-based scoring functions for evaluating RNA–protein complexes. The top approach in the figure employs chemical context profiles to represent RNA–protein complexes, followed by Sequential Forward Selection (SFS) to build a machine-learning model that reduces the initial 300-dimensional pair representation to a lower-dimensional space. The bottom approach first involves molecular docking, gridding on each nucleotide and residue, and using voxels containing atomic occupancy, mass, and charge to expand the input features. A 4D convolutional neural network is then employed to construct a machine learning model that integrates sequential and geometric dimensions. After model training, both models can score and rank RNA–protein complexes, facilitating the selection of structures with the highest probability scores.

In recent years, rapid advancements in artificial intelligence have had a profound impact on science and technology [80,81,82,83,84,85,86]. One breakthrough example is AlphaFold [41,42,43], a machine-learning-based approach that has revolutionized protein structure prediction with remarkable accuracy. While methods for predicting and modeling the 3D structures of proteins [41,42,43,87], RNAs [47,48,50], and protein–protein complexes [55,88] have made significant progress, emerging research highlights the growing efficacy of machine learning in evaluating RNA–protein complex structures, as summarized in Figure 3 and Table 3. The following sections will provide a detailed overview of these cutting-edge methods, showcasing how machine learning is reshaping our understanding and evaluation of RNA–protein interactions.

*Parisien’s potential:* In 2013, the Parisien group developed a machine-learning-based scoring function by utilizing the interface’s CCP (chemical context profile) from known RNA–protein complex structures [89] (Table 3). Specifically, the *CCP* is defined as a 300-dimensional vector:

(24) $$\vec{CCP}=\left(\sum_{C_{\beta }}^{ala}\sum_{M}^{A}f\left(r\right),\;\sum_{C_{\beta }}^{ala}\sum_{m}^{A}f\left(r\right),\sum_{C_{\beta }}^{ala}\sum_{P}^{A}f\left(r\right),\ldots ,\sum_{C_{\beta }}^{val}\sum_{P}^{T}f\left(r\right)\right)$$

Each double sum of *CCP* is the summation of the interaction strengths over a given pair. For example, the first interaction term involves the $C_{\beta }$ of alanine (Ala) and the major groove of adenosine (A), with the interaction strength set to be inversely proportional to the distance between these pairs. This scoring function employs a simplified representation of both RNA and protein structures. Specifically, the 300 dimensions are derived from 20 amino acid types and 15 nucleic acid types. For nucleic acids, a heavy atom in the major groove (M), one in the minor groove (m), and a phosphate group (P) are selected, respectively, as the interaction centers for the five nucleotides [A, C, G, U, and T], covering both RNA and DNA. In the context of RNA, entries associated with thymines (T) have a CCP value of zero. For proteins, the $C_{\beta }$ carbon atom servers as the interaction center for each residue, simplifying the complex interactions into a manageable framework while retaining essential biochemical information. $f\left(r\right)$ is the distance-dependent energy function assigned to each interaction pair:

(25) $$f\left(r\right)=\frac{1}{\max(3.5Å,r-\hat{e})}$$

where *r* is the distance between the interaction centers, and $\hat{e}$ is the average distance between $C_{\beta }$ and its partner interaction center. Any RNA–protein complex is represented with the CCP vector. The similarity of the decoy and the native complexes can be obtained by computing the angle between their CCP vectors. This angle, or CCD (chemical context discrepancy), is defined as a relation in terms of two arbitrary vectors $\vec{{CCP}_{1}}$ and $\vec{{CCP}_{2}}$:

(26) $$\cos(CCD)=\frac{\left(\vec{{CCP}_{1}}\cdot \vec{{CCP}_{2}}\right)}{\left(\left|\vec{{CCP}_{1}}\right|\times \left|\vec{{CCP}_{2}}\right|\right)}$$

The more different the CCPs, which represent the chemical properties of the RNA–protein complex interface, the greater the angle. Then, the CCP-based scoring function *S* is designed by weighting the entries of a CCP to identify near-native structures with low *CCD* values to the native structure:

(27) $$S=Coulomb+\vec{\omega_{ccp}}\cdot \vec{ccp}$$

where Coulomb is the generic electrostatic energy term, and $\vec{\omega_{ccp}}$ is a vector enabling the weighted sum of *CCP* components. The forward version of the sequential feature selection (SFS) approach, a machine-learning-based method, is used to identify the most important interacting pairs among all possible ones, reducing the nonzero entries in $\vec{\omega_{ccp}}$ to 12 from the original 300 dimensions [90]. After training, these dimensions are further reduced to 12 for scoring tRNA–protein complexes and 6 for scoring other RNA–protein complexes. This potential is not available as a standalone program.

*DRPScore:* In 2023, the Zhao group developed a deep-learning-based scoring function, DRPScore, to better account for the structural flexibility of RNA–protein complexes [23]. Specifically, DRPScore utilizes a 4D convolutional neural network to train models that can effectively identify near-native RNA–protein structures. To overcome the limitations of scarce data, DRPScore utilizes physics-based simulations targeting RNA–protein interfaces, generating 500 decoy structures for each RNA–protein complex in the initial process. This approach enabled the creation of a training dataset with over 100,000 structures, a significant improvement over the typical dataset size of fewer than 300 structures in traditional knowledge-based methods. The input for DRPScore includes nucleotides and residues at the RNA–protein interaction interface within a 6 Å distance. The model accurately describes molecular systems at the atomic level, classifying 85 atom types for RNA nucleotides and 225 atom types for protein residues. It assigns accurate mass and charge values to each atom through detailed feature processing. The 4D convolutional approach includes an additional operation along the sequential dimension, preserving critical information about nucleotide and residue interactions.

During preprocessing, each RNA–protein complex is represented as a tensor with the dimensions 1×3×L×(H×W×D). Here, the value 3 corresponds to the three captured features: the accumulations of the occupation number, mass, and charge of the atoms within each grid box. The parameter *L* = 128 represents the maximum allowable length for RNA–protein complex sequences, while *H*, *W*, and *D* define the height, width, and depth of a 3D cube that represents each nucleotide and residue within the RNA–protein complex, with each dimension set to 32 units. This grid structure effectively captures the spatial arrangement and intricate interactions at the atomic level, providing a detailed representation of the molecular architecture and facilitating accurate modeling of RNA–protein interactions.

DRPScore comprises six layers, with the final layer being a fully connected layer for classification. Each of the first five layers includes a Conv4d module, an optional BatchNorm module, and a MaxPooling module. In these Conv4d modules, the channel numbers progressively change: 64, 128, 256, 512, and 512. The strides applied in each module are set to 2, 2, 2, 1, and 1, respectively. This design effectively halves the feature length of the RNA–protein complex in the first three blocks while maintaining it in the last two blocks. All MaxPooling layers have a kernel size and stride of 2, halving each pooling module’s height, width, and depth dimensions. The final representation of the RNA–protein complex is obtained through global average pooling, resulting in an 8192-dimensional feature vector that encapsulates the complex’s characteristics. Finally, after applying a 4D convolution in the last layer, an adaptive spatial pooling for the final RNA–protein complex representation $O_{overall}$ is utilized:

(28) $$O_{overall}=\frac{1}{H}\frac{1}{W}\frac{1}{D}\sum_{i\in H}\sum_{i\in W}\sum_{i\in D}O_{LN}\;[i,j,k]$$

After adding a linear classification layer to the model, probability scores can be generated to evaluate and select near-native RNA–protein complex structures. The representations learned by DRPScore effectively capture intra- (local) and inter-nucleotide/residue (global) information. This is accomplished by integrating convolutional layers along the sequence dimension, while also expanding on the spatial dimension. Each layer progressively models a wider range of interactions between nucleotides and residues. It has been extensively evaluated for its ability to identify near-native RNA–protein structures across diverse cases. Although DRPScore achieves comparable success in bound–bound cases and outperforms knowledge-based methods in more challenging unbound–unbound cases, its success rate in these unbound–unbound cases still requires substantial improvement. Recently, this method has also been successfully extended to evaluate the structure of DNA–protein complexes [91]. The package of the model is freely available at https://github.com/Zhaolab-GitHub/DRPScore_v1.0 (accessed on 29 September 2024).

## 4. Benchmarks and Datasets for Assessing Scoring Functions

Evaluating scoring functions for RNA–protein complexes requires rigorous testing on a 3D RNA–protein complex structural benchmark. Consequently, various benchmarks and datasets have been established to assess performance. This section discusses the multiple benchmarks curated by different groups (Table 4). Due to the inherent flexibility of both RNA and proteins, significant conformational changes can be induced during the docking process. The RNA–protein complexes in these datasets can be broadly categorized into three types: (1) bound–bound cases, (2) bound–unbound cases, and (3) unbound–unbound cases [92,93,94].

In bound–bound cases, no conformational changes occur in the RNA or the protein during the docking process. This means that both monomers involved in the docking come from the same complex. Bound–unbound cases unequivocally involve conformational changes in the RNA or the protein, where one may exist in an unbound state or come from a different complex. Unbound–unbound cases explicitly refer to scenarios where both the RNA and protein are in unbound conformations or originate from two distinct complexes. These scenarios undeniably make the docking process particularly challenging due to the complex conformational shifts involved.

For a robust performance evaluation, benchmark datasets must possess three key characteristics: (1) Diversity of targets. The benchmarks must include a wide range of targets to effectively test the robustness of different molecular docking algorithms. (2) Experimentally resolved structures: It is crucial to use datasets derived from experimentally resolved structures to avoid introducing computational biases or errors. (3) Bound and unbound conformations: The benchmarks must contain both bound and unbound conformations of the individual monomers, allowing for the assessment of conformational changes upon complex formation [96,97].

Benchmark I constructed by Zou et al. comprises 72 RNA–protein complexes, among which 52 are unbound–unbound cases and 20 are bound–unbound cases [95]. Based on the degree of conformational change observed in unbound structures upon binding, these 72 RNA–protein complexes can be further categorized into 49 easy ($I_{rmsd}\leq 1.5\;Å$ or $f_{nat}\geq 0.8$), 16 medium ($1.5\;Å<I_{rmsd}\leq 4.0\;Å$ and $0.4<f_{nat}\leq 0.8$), and 7 difficult targets ($I_{rmsd}>4.0\;Å$ or $f_{nat}<0.4$). The interface root mean square deviation ($I_{rmsd}$) is defined as the RMSD of the interaction interface region after optimal superimposition of the bound and unbound conformations. The fraction of native contacts ($f_{nat}$) is defined as the proportion of native nucleotide–residue pairs in the unbound conformation. Specifically, it is the ratio of the number of native nucleotide–residue pairs in the optimally superimposed unbound conformation to the total number of nucleotide–residue pairs in the bound conformation. The RNA–protein complex benchmark can be accessed at https://zoulab.dalton.missouri.edu/RNAbenchmark/index.htm (accessed on 29 September 2024).

Benchmark II constructed by Fernandez-Recio et al. comprises 106 RNA–protein complexes [93]. Among these cases, 71 cases were taken from crystallography or NMR experiments, while 35 cases were built using homology modeling. Of the 71 experimental RNA–protein complexes, 9 unbound–unbound cases and 62 bound–unbound cases exist. The 35 homology-modeled cases consist of 13 unbound–model, 19 bound–model, and 3 model–model RNA–protein complexes. In unbound–model cases, the RNA or protein exists in an unbound state or comes from a different complex, while the other is a homology-based prediction structure. In bound–model cases, one molecule is in a bound state, and the other is a homology-based prediction structure. In model–model cases, both the RNA and protein are homology-based prediction structures, with no native complex involved. Based on the degree of conformational change observed in unbound structures upon binding, these 106 RNA–protein complexes can also be further categorized into 64 easy ($0\leq I_{rmsd}<2.5\;Å$), 24 medium ($2.5\leq I_{rmsd}\leq 5.0\;Å$), and 18 difficult ($I_{rmsd}>$ 5.0 Å) targets. The RNA–protein complexes benchmark can be accessed at https://life.bsc.es/pid/protein-rna-benchmark/ (accessed on 29 September 2024).

Benchmark III constructed by Bahadur et al. comprises 45 RNA–protein complexes, among which 9 are unbound–unbound cases and 36 are bound–unbound cases [92]. Based on the degree of conformational change observed in unbound structures upon binding, these 45 RNA–protein complexes can also be further categorized into 34 easy ($0\leq I_{rmsd}<1.5\;Å$), 8 medium ($1.5\leq I_{rmsd}<3.0\;Å$), and 3 difficult ($I_{rmsd}\geq$ 3.0 Å). Later, Bahadur et al. developed an extended version of benchmark III [94]. The non-redundant RNA–protein complex benchmark contains 126 RNA–protein complexes, a three-fold increase in the number of structures compared to the previously proposed RNA–protein complex benchmark III. Among these cases, 21 are unbound–unbound cases and 105 are bound–unbound cases. Also, based on the degree of conformational change observed in unbound structures upon binding, these 126 RNA–protein complexes can also be further categorized into 72 easy ($0\leq I_{rmsd}<1.5\;Å$), 25 medium ($1.5\leq I_{rmsd}<3.0\;Å$), and 19 difficult ($I_{rmsd}\geq$ 3.0 Å).

## 5. Criteria and Assessment of the Prediction Quality

The quality of RNA–protein complex predictions is evaluated using the CAPRI criteria [98,99], focusing on two primary metrics: interface root mean square deviation ($I_{rmsd}$) and ligand root mean square deviation ($L_{rmsd}$). $I_{rmsd}$ measures the deviation at the interface between native and predicted structures after protein superposition. $L_{rmsd}$ quantifies the displacement of the RNA under the same conditions. Specifically, interface residues and nucleotides are extracted from both the native RNA–protein complexes and the decoys, and superposition is performed to calculate $I_{rmsd}$. Similarly, all nucleotides from native and decoy structures are extracted and superposed to calculate $L_{rmsd}$. This ensures an accurate assessment of deviations at the interaction interface and the overall RNA conformation [100]. Typically, a decoy is classified as a near-native structure if its $I_{rmsd}$ relative to the native complex is ≤4.0 Å, or if its $L_{rmsd}$ is ≤10.0 Å. A scoring function is successful if it ranks near-native structures among the top N decoys [22]. The root mean square deviation (including $I_{rmsd}$ and $L_{rmsd}$) is defined as

(29) $$RMSD=\sqrt{\frac{1}{N}\sum_{i}\left({\left|\vec{X_{A_{i}}}-\vec{X_{B_{i}}}\right|}^{2}+{\left|\vec{Y_{A_{i}}}-\vec{Y_{B_{i}}}\right|}^{2}+{\left|\vec{Z_{A_{i}}}-\vec{Z_{B_{i}}}\right|}^{2}\right)}$$

where $\vec{X}$, $\vec{Y}$, and $\vec{Z}$ represent the coordinates of the native and predicted structures. *N* is the total number of atoms.

The evaluation of RNA–protein structures is an area of research that has not been extensively explored. Previous studies have mainly focused on rigid-body docking, overlooking the structural flexibility inherent in RNA–protein interactions. Although scoring functions in rigid-body docking have achieved success rates of about 80%, they still need significant improvement for flexible docking scenarios, especially in fully flexible unbound–unbound docking. One major challenge in this field is accurately sampling the dynamic conformations that RNAs and proteins adopt during their interactions. In fully flexible unbound–unbound docking, the interaction interface can change dramatically, which presents a significant challenge to scoring functions that have not previously encountered such diverse structures.

**Figure 4:**
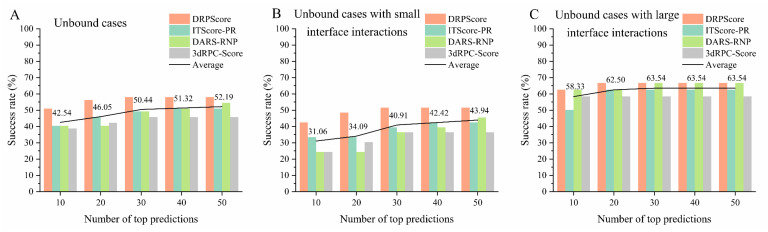
The performance of various scoring functions on the unbound cases from RNA–protein complex benchmark I. The success rate of DRPScore (orange bar), ITScore-PR (blue bar), DARS-RNP (green bar), 3dRPC-Score (gray bar), and the average (black line) on the (**A**) unbound cases, (**B**) unbound cases with small interface interactions, and (**C**) unbound cases with extensive interface interactions from RNA–protein complex benchmark I.

As shown in Figure 4A, we evaluated the performance of various scoring functions on the most challenging unbound cases from RNA–protein complex benchmark I. The benchmark comprises 57 unbound cases, selected using a 0.95 sequence similarity cutoff, using CD-HIT to avoid redundancy [101,102,103]. The assessment focused on three types of scoring functions: coarse-grained knowledge-based methods (e.g., DAR-RNP and 3dRPC-Score), all-atom knowledge-based methods (e.g., ITScore-PR), and recent machine-learning-based methods (e.g., DRPScore). Among these, the machine-learning-based DRPScore consistently outperformed the traditional scoring functions. Specifically, DRPScore achieved the highest success rates across all prediction categories, reaching a peak of 57.89% for the top 10, 20, 30, 40, and 50 predictions. This result underscores the superiority of machine learning approaches in evaluating RNA–protein complex structures more accurately than conventional methods. However, despite the advancements represented by these scoring functions, the overall success rates across all methods remain below 60%, averaging 52.19% for the top 50 predictions. This limitation highlights the need for significant refinements in current approaches to achieve a higher accuracy in predicting RNA–protein interactions. These findings also indicate a need for developing more sophisticated models or integrating additional biological data to improve the accuracy of these tools, especially in complex conformational change docking scenarios. The detailed performance and corresponding PDB IDs are provided in Supplementary Tables S1 and S2.

Moreover, we further evaluated the performance of various scoring functions on the unbound cases with different interface interactions from RNA–protein complex benchmark I. We initially calculated the interaction interface of the RNA–protein complex using a 6 Å distance cutoff. The number of nucleotides and amino acids at the interaction interface ranged from a minimum of 23 to a maximum of 199, with an average of 75. Therefore, we categorized interactions based on the number of amino acids and nucleotides at the interface, using 70 as the threshold to distinguish between relatively small and large interface interactions. Figure 4B shows that the machine-learning-based DRPScore consistently outperformed traditional scoring functions for cases involving relatively small interface interactions. Moreover, Figure 4C shows that the success rates were slightly improved compared to those in small interface interactions for cases involving relatively large interface interactions. Even in the top 10 predictions, the average success rate for these methods reached 58.33%. However, the overall success rates across all methods remained relatively low, with the highest average success rate in the top 50 being just 63.54%. The detailed performance and corresponding PDB IDs are provided in Supplementary Tables S3–S6.

The results highlight the importance of interaction interface size in the performance of scoring functions for predicting RNA–protein complexes. While machine-learning-based approaches like DRPScore outperform traditional scoring methods in smaller interaction interfaces, there is still room for improvement in overall success rates. The superior performance of machine-learning-based methods is due to their ability to capture complex multi-body interactions at the interface, which traditional methods often miss. However, the limited information within smaller interfaces restricts the model’s ability to learn and score interactions accurately. Additionally, smaller interfaces often have more structural flexibility and involve complex non-specific interactions, making precise modeling and prediction more challenging. Success rates improve with more extensive interaction interfaces. This is due to the increased contact points and features within these interfaces, allowing models to capture key interaction patterns more effectively and enhance prediction accuracy.

**Figure 5:**
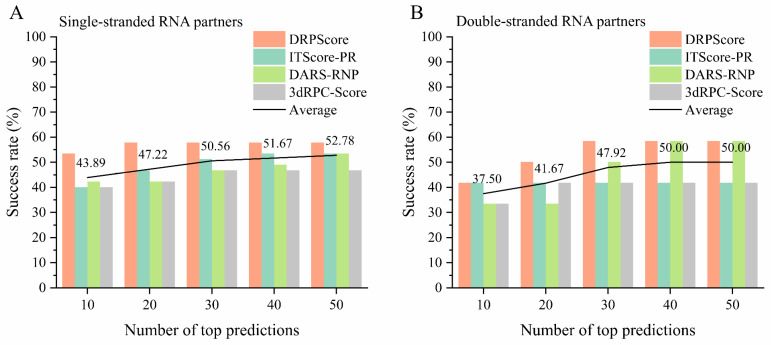
The performance of various scoring functions on the unbound cases from RNA–protein complex benchmark I. The success rate of DRPScore (orange bar), ITScore-PR (blue bar), DARS-RNP (green bar), 3dRPC-Score (gray bar), and average (black line) on the (**A**) single-stranded RNA partners, and (**B**) double-stranded RNA partners from RNA–protein complex benchmark I.

We further evaluated the performance of various scoring functions on unbound cases involving either single-stranded or double-stranded RNA partners from RNA–protein complex benchmark I. As shown in Figure 5A, the machine-learning-based DRPScore consistently outperformed traditional scoring functions in cases with single-stranded RNA partners. Figure 5B demonstrates a slight improvement in success rates compared to cases with double-stranded RNA partners. Overall, the performance of all scoring functions was lower in cases involving double-stranded RNA partners. In the top 10 predictions, the average success rate of each scoring function for single-stranded RNA cases was 43.89%, compared to 37.50% for double-stranded RNA cases. This discrepancy may be attributed to the increased complexity of multi-body interactions at the RNA–protein interface. In the case of double-stranded RNA, interactions involve both the protein and the inter-chain interactions between the two RNA strands. These additional layers of complexity make it more challenging for scoring functions to model the binding interface accurately. The detailed performance and corresponding PDB IDs are provided in Supplementary Tables S7–S10.

## 6. Discussion and Future Directions

**Figure 6:**
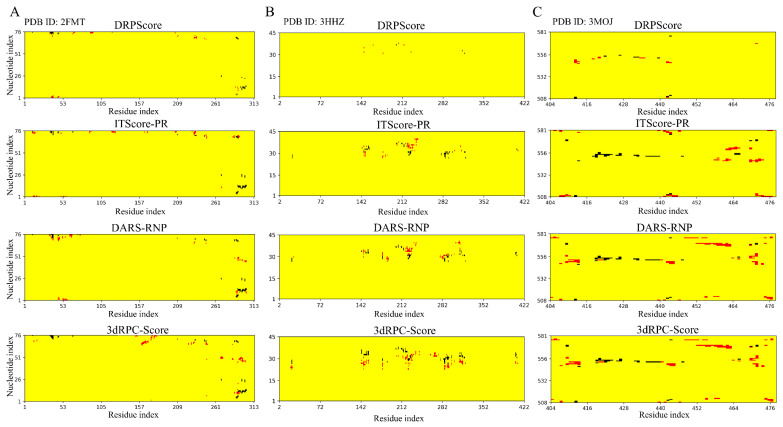
Contact distributions for three unbound docking examples. The contact maps (from top to bottom) show interactions between nucleotides and residues within a 6 Å range in the lowest RMSD model among the top 10 models selected by DRPScore, ITScore-PR, DARS-RNP, and 3dRPC-Score for (**A**) PDB ID: 2FMT, (**B**) PDB ID: 3HHZ, and (**C**) PDB ID: 3MOJ. Red and black dots indicate nucleotide–residue pairs that are added or reduced in the models selected by each scoring function compared to the native RNA–protein complex structure.

Predicting the structure of RNA–protein complexes is essential for understanding biological processes and developing new treatments. Several factors influence local interactions in these complexes. Figure 6 shows the differences in structural selections made by each scoring function across three examples, highlighting their respective abilities to capture local interaction features. The analysis focuses on nucleotide–residue pairs within a cutoff distance of 6 Å compared to the native structures. The red and black dots represent nucleotide–residue pairs that are added or reduced relative to the native RNA–protein complex structure. Overall, each scoring function captures native interactions to different extents, with changes primarily occurring in localized regions. DDPScore captures interactions closely aligned with the native contacts, indicating minimal disruption. Similarly, ITScore-PR also captures interactions near the native positions, albeit to a slightly lesser degree. In contrast, DARS-RNP and 3dRPC-Score identify interactions that deviate further from the native contacts. This discrepancy may be due to the reliance of DARS-RNP and 3dRPC-Score on a coarse-grained representation, which simplifies molecular details and omits crucial side-chain interaction information. Since side-chain interactions play a crucial role in determining the specificity and strength of RNA–protein binding, these models may struggle to capture the nuanced geometric and energetic properties necessary for precise structural predictions. On the other hand, ITScore-PR, which counts atom–atom pairs, can capture more precise atom-level interactions. The machine-learning-based scoring function DRPScore efficiently recognizes local features of RNA–protein complex structures. This capability enables it to accurately capture binding patterns and effectively distinguish structures resembling the native state.

**Figure 7:**
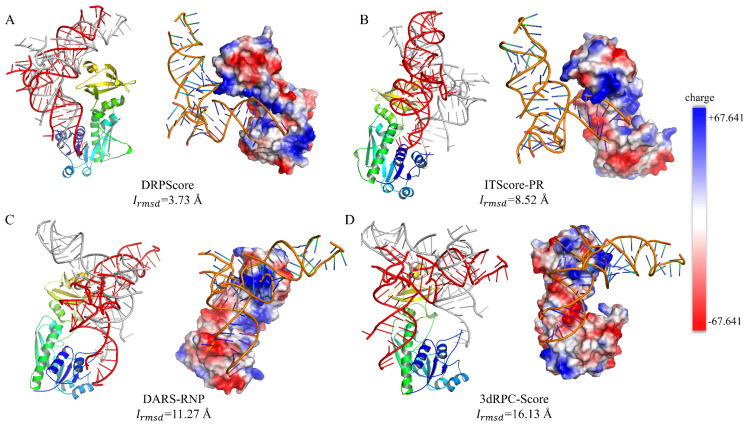
The lowest interface RMSD model and electrostatic interaction distribution. The lowest interface RMSD model and the corresponding electrostatic interaction distribution among the top 10 models (RNA in red) selected by (**A**) DRPScore, (**B**) ITScore-PR, (**C**) DARS-RNP, and (**D**) 3dRPC-Score, compared to the native RNA–protein complex (RNA in gray). Positively charged regions are shown in blue, while negatively charged regions are shown in red.

Specifically, we utilized methionyl-tRNAfMet formyltransferase complexed with formyl-methionyl-tRNAfMet (PDB ID: 2FMT) to analyze the electrostatic interactions between RNA and protein using PyMOL (version 1.8.0.3). Figure 7 shows the lowest interface RMSD model among the top 10 models selected by each scoring function. The lowest $I_{rmsd}$ for the DRPScore-selected model is 3.73 Å, compared to 8.52 Å for ITScore-PR, 11.27 Å for DARS-RNP, and 16.13 Å for 3dRPC-Score. Overall, the RNA in the selected structures tends to bind to similar regions on the protein, likely due to intrinsic properties of the protein surface, such as electrostatic potential and hydrophobicity, which naturally favor specific binding sites. However, the varying RMSD values suggest geometric matching and positioning accuracy differences. DRPScore effectively captures the interface interaction patterns and achieves a lower $I_{rmsd}$. In contrast, scoring functions like DARS-RNP and 3dRPC-Score show higher deviations, possibly due to their reliance on coarse-grained representations, which may lack the precision to accurately model spatial alignment and side-chain interactions essential for RNA–protein binding. ITScore-PR, with its moderate performance, balances these aspects but still falls short in capturing the intricate details of the RNA–protein interface. Since RNA carries a strong negative charge, it is expected to bind preferentially to the positively charged regions of proteins. The DRPScore-selected model shows RNA bound to a positively charged protein region. The model selected by ITScore-PR primarily binds to a positively charged region, with only minor deviations from favorable electrostatic interactions. In contrast, structures selected by DARS-RNP and 3dRPC-Score often bind to negatively charged regions, indicating less accurate electrostatic complementarity. This discrepancy may be attributed to DRPScore explicitly incorporating atomic charge information as input features during training, enabling it to capture electrostatic interactions precisely. Since the net charge of a protein is primarily distributed on its side chains, all-atom knowledge-based scoring functions can capture more detailed interaction features compared to coarse-grained scoring functions. Considering these findings, future advancements in scoring functions should focus on developing methods tailored to the specific characteristics of various interaction interfaces. A particular emphasis should be placed on accurately modeling complex multi-body interactions, to enhance prediction robustness and precision.

Recently, there has been a growing focus on predicting the structures of RNA–protein complexes in biomolecular research. This interest has been fueled by advancements in machine learning techniques, which have significantly improved structure prediction [39,43,104]. The increased interest in this area has led to significant progress in predicting the structures of individual structures and complex predictions like protein–protein and RNA–protein complexes.

This review has compared two main scoring functions for predicting RNA–protein complexes: knowledge-based (coarse-grained and all-atom) and machine-learning-based approaches. While each scoring function has its advantages, both types of scoring functions share a common limitation: they lack a strong theoretical foundation in physics. For example, knowledge-based scoring functions often assess RNA–protein interactions using a weighted sum of statistical potentials. However, the exact relationship between these scores and the system’s free energy needs to be defined. Applying machine-learning-based scoring functions in evaluating RNA–protein complexes has shown promise. These functions outperform traditional methods by capturing complex, multibody interactions in RNA–protein binding. They can learn from vast amounts of data and represent intricate interaction patterns that are difficult for traditional methods. However, they have a relatively low success rate in challenging unbound–unbound cases, typically below 60%. These models struggle with structural diversity within training datasets, leading to potential overfitting. Integrating knowledge-based models could help mitigate these issues and enhance prediction accuracy.

One of the major challenges in predicting RNA–protein interactions is the conformational flexibility of both RNA and protein components upon binding. For example, in the NF-κB dimer system, the RMSD of RNA before and after binding can be as high as 5.4 Å [68]. Although extensive docking simulations can generate large datasets to address data scarcity, accurately modeling the loose atomic packing and unique interactions remains a challenge. A combination of various docking and scoring methodologies can be used to develop consensus models, clustering predictions based on their scoring outcomes to enhance reliability. In cases where a consensus is not achieved, the top-scoring models from different methods could be proposed as alternative solutions. Knowledge-based statistical potentials are effective for rigid structures and large interaction interfaces. However, for cases with small interaction interfaces or complex flexible structures, deep learning approaches may be required to accurately capture the intricate multi-body interactions.

Current machine-learning-based scoring functions are hindered by the lack of three-dimensional structural data and the highly variable nature of RNA–protein interfaces. A promising method to enhance RNA–protein complex prediction involves integrating multi-scale modeling techniques, which combine coarse-grained and all-atom models to address the diverse nature of RNA–protein interfaces at different resolutions. This multi-stage approach allows for the rapid identification of potential conformations using coarse-grained models, then refined and precisely scored with all-atom models. The approach achieves detailed structural information and accommodates conformational changes. Moreover, developing dynamic scoring functions that adjust weights based on the local environment of RNA and proteins could provide greater flexibility in handling conformational changes, especially in regions with loosely packed atoms at the interface. Such approaches leverage the strengths of different scales, capturing relationships and features that single-modal methods might miss. Additionally, integrating high-resolution structural data from crystallography or NMR, low-resolution information from cryo-electron microscopy, or other experimental techniques could enhance the robustness of RNA–protein complex predictions [88,105,106,107].

RNA–protein complex prediction remains a challenge in the fields of soft matter physics and biophysics. With advancing computational techniques, structure prediction becomes a potent complement to experimental methods, offering fresh insights into the RNA–protein mechanism and downstream applications. Despite persistent challenges, particularly in flexible docking and complex assembly, the ongoing advancements in experimental and computational approaches are poised to drive transformative breakthroughs imminently.

## Figures and Tables

**Table 1:** List of coarse-grained knowledge-based scoring methods for RNA–protein complex structure evaluation. This table includes the development time, the representation of RNA–protein molecules, the type and features of these methods, and their availability.

Name	Time	Feature	Availability as a Standalone Method	Reference
Fernández’s potential	2010	Pairwise nucleotide–residue propensity	N/A	[63]
DARS-RNP	2011	Decoys as the reference state potential	https://genesilico.pl/software/stand-alone/statistical-potentials (accessed on 29 September 2024)	[36]
QUASI-RNP	2011	Quasi-chemical potential	https://genesilico.pl/software/stand-alone/statistical-potentials (accessed on 29 September 2024)	[36]
Zacharias’s potential	2011	Distance-dependent potential	N/A	[71]
Wang’s potential	2012	Pairwise nucleotide–residue propensity with secondary information	N/A	[72]
Deck-RP	2013	Distance- and environment-dependent potential	http://biophy.hust.edu.cn/new/3dRPC (accessed on 29 September 2024)	[69]
RPRANK	2016	Pairwise nucleotide–residue propensity; RMSD	http://biophy.hust.edu.cn/new/3dRPC (accessed on 29 September 2024)	[70]
3dRPC-Score	2017	Conformations of nucleotide–residue pairs	http://biophy.hust.edu.cn/new/3dRPC (accessed on 29 September 2024)	[37]

**Table 2:** List of all-atom knowledge-based scoring methods for RNA–protein complex structure evaluation. This table includes the development time, the representation of RNA–protein molecules, the type and features of these methods, and their availability.

Name	Time	Feature	Availability as a Standalone Method	Reference
Varani’s H-bonding potential	2004	Hydrogen-bonding potential	N/A	[11]
Varani’s all-atom potential	2007	Distance-dependent potential	N/A	[76]
dRNA	2011	Volume-fraction corrected distance-scaled, finite, ideal gas reference (DFIRE) energy function	N/A	[12]
ITScore-PR	2014	Pairwise distance-dependent potential; iterative	https://zoulab.dalton.missouri.edu/resources_itscorepr.html (accessed on 29 September 2024)	[22]
DITScore-PR	2019	Pairwise distance-dependent potential; double-iterative	http://huanglab.phys.hust.edu.cn/mprdock/ (accessed on 29 September 2024)	[31]

**Table 3:** List of machine-learning-based scoring methods for RNA–protein complex structure evaluation. This table includes the development time, the representation of RNA–protein molecules, the type and features of these methods, and their availability.

Name	Time	Representation	Feature	Availability as a Standalone Method	Reference
Parisien’s potential	2013	Coarse-grained	Chemical context profiles	N/A	[89]
DRPScore	2023	All-atom	Convolutional neural network	https://github.com/Zhaolab-GitHub/DRPScore_v1.0 (accessed on 29 September 2024)	[23]

**Table 4:** List of RNA–protein complex docking benchmarks. The time and number of total, bound–unbound, unbound–unbound, easy, medium, and difficult cases are listed in this table.

Benchmark	Development	Time	Total Number of Cases	Number of Cases						Availability
				Bound–Unbound	Unbound–Unbound	Easy	Medium	Difficult	Reference	
Benchmark I	Zou group	2013	72	20	52	49	16	7	[95]	https://zoulab.dalton.missouri.edu/RNAbenchmark/index.htm (accessed on 29 September 2024)
Benchmark II	Fernández-Recio group	2012	106 *	62	9	64	24	18	[93]	https://life.bsc.es/pid/protein-rna-benchmark/ (accessed on 29 September 2024)
Benchmark III	Bahadur group	2012/2016	126	105	21	72	25	19	[92,94]	N/A

* Contains 35 homology-modeled cases.

## Supplementary Materials

The following supporting information can be downloaded at: https://www.mdpi.com/article/10.3390/biom14101245/s1. Table S1: The performance of DRPScore, ITScore-PR, DARS-RNP, and 3dRPC-Score on unbound cases in RNA-protein complex benchmark I; Table S2: The PDB IDs of the unbound cases in RNA-protein complex benchmark I for the performance of DRPScore, ITScore-PR, DARS-RNP, and 3dRPC-Score; Table S3: The performance of DRPScore, ITScore-PR, DARS-RNP, and 3dRPC-Score on unbound cases with relatively small interface interactions in RNA-protein complex benchmark I; Table S4: The PDB IDs of the unbound cases with relatively small interface interactions in RNA-protein complex benchmark I for the performance of DRPScore, ITScore-PR, DARS-RNP, and 3dRPC-Score; Table S5: The performance of DRPScore, ITScore-PR, DARS-RNP, and 3dRPC-Score on unbound cases with relatively large interface interactions in RNA-protein complex benchmark I; Table S6: The PDB IDs of the unbound cases with relatively large interface interactions in RNA-protein complex benchmark I for the performance of DRPScore, ITScore-PR, DARS-RNP, and 3dRPC-Score; Table S7: The performance of DRPScore, ITScore-PR, DARS-RNP, and 3dRPC-Score on unbound cases with single-stranded RNA partners in RNA-protein complex benchmark I; Table S8: The PDB IDs of the unbound cases with relatively large interface interactions in RNA-protein complex benchmark I for the performance of DRPScore, ITScore-PR, DARS-RNP, and 3dRPC-Score; Table S9: The performance of DRPScore, ITScore-PR, DARS-RNP, and 3dRPC-Score on unbound cases with double-stranded RNA partners in RNA-protein complex benchmark I; Table S10: The PDB IDs of the unbound cases with relatively large interface interactions in RNA-protein complex benchmark I for the performance of DRPScore, ITScore-PR, DARS-RNP, and 3dRPC-Score.
