# Supplementary material for: Advances and Challenges in Scoring Functions for RNA–Protein Complex Structure Prediction
Source: Biomolecules. 2024 Oct 1;14(10):1245. doi: 10.3390/biom14101245 (PMC11506084; doi:10.3390/biom14101245)
Supplement: Supplementary file 1 [file biomolecules-14-01245-s001.zip › biomolecules-3217953-supplementary.pdf]

Supplementary Information:

Advances and Challenges in Scoring Functions for RNA–Protein Complex Structure Prediction

Chengwei Zeng†, Chen Zhuo†, Jiaming Gao, Haoquan Liu and Yunjie Zhao*

Institute of Biophysics and Department of Physics, Central China Normal University, Wuhan 430079, China;
cwzengwuhan@mails.ccnu.edu.cn (C.Z.); chenzhuowh@mails.ccnu.edu.cn (C.Z.); jmgao@mails.ccnu.edu.cn (J.G.); liuhaoquan@mails.ccnu.edu.cn (H.L.)
* Correspondence: yjzhaowh@ccnu.edu.cn
† These authors contributed equally to this work.

Supplementary Table S1. The performance of DRPScore, ITScore-PR, DARS-RNP, and 3dRPC-Score on unbound cases in RNA-protein complex benchmark I.

Number of top predictions	Success rate (%)				
	Machine learning-based	All-atom knowledge-based	Coarse-grained knowledge-based		Average
	DRPScore	ITScore-PR	DARS-RNP	3dRPC-Score	
10	50.88	40.35	40.35	38.60	42.54
20	56.14	45.61	40.35	42.11	46.05
30	57.89	49.12	49.12	45.61	50.44
40	57.89	50.88	50.88	45.61	51.32
50	57.89	50.88	54.39	45.61	52.19

Supplementary Table S2. The PDB IDs of the unbound cases in RNA-protein complex benchmark I for the performance of DRPScore, ITScore-PR, DARS-RNP, and 3dRPC-Score.

1T0K, 3LRR, 2ANR, 1J1U, 1YVP, 2UWM, 3DD2, 3MOJ, 2AKE, 2DU3, 1DFU, 1JID, 1E8O, 1JBS, 1H3E, 1RC7, 1S03, 2FMT, 2XDB, 2CZJ, 2QUX, 1SER, 2VPL, 1Q2R, 1G1X, 1H4S, 1UN6, 1R9F, 2AZ0, 3HHZ, 1OOA, 1MMS, 3CIY, 2CSX, 3ADD, 1KOG, 2RFK, 2HW8, 2R8S, 3OVB, 2GJW, 1C0A, 3FOZ, 2ZM5, 2BH2, 2ZNI, 2BTE, 1IL2, 3EPH, 2ZZM, 1F7U, 2ZUE, 1QTQ, 1N78, 3LWR, 1GAX, 2IPY

Supplementary Table S3. The performance of DRPScore, ITScore-PR, DARS-RNP, and 3dRPC-Score on unbound cases with relatively small interface interactions in RNA-protein complex benchmark I.

Number of top predictions	Success rate (%)				
	Machine learning-based	All-atom knowledge-based	Coarse-grained knowledge-based		Average
	DRPScore	ITScore-PR	DARS-RNP	3dRPC-Score	
10	42.42	33.33	24.24	24.24	31.06
20	48.48	33.33	24.24	30.30	34.09
30	51.52	39.39	36.36	36.36	40.91
40	51.52	42.42	39.39	36.36	42.42
50	51.52	42.42	45.45	36.36	43.94

Supplementary Table S4. The PDB IDs of the unbound cases with relatively small interface interactions in RNA-protein complex benchmark I for the performance of DRPScore, ITScore-PR, DARS-RNP, and 3dRPC-Score.

1T0K, 3LRR, 2ANR, 1J1U, 1YVP, 2UWM, 3DD2, 3MOJ, 2AKE, 2DU3, 1DFU, 1JID, 1E8O, 1JBS, 1H3E, 1RC7, 1S03, 2FMT, 2XDB, 2CZJ, 2QUX, 1SER, 2VPL, 1Q2R, 1G1X, 1H4S, 1UN6, 1R9F, 2AZ0, 3HHZ, 1OOA, 1MMS, 3CIY

Supplementary Table S5. The performance of DRPScore, ITScore-PR, DARS-RNP, and 3dRPC-Score on unbound cases with relatively large interface interactions in RNA-protein complex benchmark I.

Number of top predictions	Success rate (%)				
	Machine learning-based	All-atom knowledge-based	Coarse-grained knowledge-based		Average
	DRPScore	ITScore-PR	DARS-RNP	3dRPC-Score	
10	62.50	50.00	62.50	58.33	58.33
20	66.67	62.50	62.50	58.33	62.50
30	66.67	62.50	66.67	58.33	63.54
40	66.67	62.50	66.67	58.33	63.54
50	66.67	62.50	66.67	58.33	63.54

Supplementary Table S6. The PDB IDs of the unbound cases with relatively large interface interactions in RNA-protein complex benchmark I for the performance of DRPScore, ITScore-PR, DARS-RNP, and 3dRPC-Score.

2CSX, 3ADD, 1KOG, 2RFK, 2HW8, 2R8S, 3OVB, 2GJW, 1C0A, 3FOZ, 2ZM5, 2BH2, 2ZNI, 2BTE, 1IL2, 3EPH, 2ZZM, 1F7U, 2ZUE, 1QTQ, 1N78, 3LWR, 1GAX, 2IPY

Supplementary Table S7. The performance of DRPScore, ITScore-PR, DARS-RNP, and 3dRPC-Score on unbound cases with single-stranded RNA partners in RNA-protein complex benchmark I.

Number of top predictions	Success rate (%)				
	Machine learning-based	All-atom knowledge-based	Coarse-grained knowledge-based		Average
	DRPScore	ITScore-PR	DARS-RNP	3dRPC-Score	
10	53.33	40.00	42.22	40.00	43.89
20	57.78	46.67	42.22	42.22	47.22
30	57.78	51.11	46.67	46.67	50.56
40	57.78	53.33	48.89	46.67	51.67
50	57.78	53.33	53.33	46.67	52.78

Supplementary Table S8. The PDB IDs of the unbound cases with relatively large interface interactions in RNA-protein complex benchmark I for the performance of DRPScore, ITScore-PR, DARS-RNP, and 3dRPC-Score.

2ANR, 1J1U, 2UWM, 3DD2, 3MOJ, 2AKE, 2DU3, 1JID, 1E8O, 1JBS, 1H3E, 1S03, 2FMT, 2XDB, 2CZJ, 2QUX, 1SER, 2VPL, 1Q2R, 1H4S, 1UN6, 3HHZ, 1OOA, 1MMS, 2CSX, 3ADD, 1KOG, 2HW8, 2R8S, 3OVB, 1C0A, 3FOZ, 2ZM5, 2BH2, 2ZNI, 2BTE, 1IL2, 3EPH, 2ZZM, 1F7U, 2ZUE, 1QTQ, 1N78, 1GAX, 2IPY

Supplementary Table S9. The performance of DRPScore, ITScore-PR, DARS-RNP, and 3dRPC-Score on unbound cases with double-stranded RNA partners in RNA-protein complex benchmark I.

Number of top predictions	Success rate (%)				
	Machine learning-based	All-atom knowledge-based	Coarse-grained knowledge-based		Average
	DRPScore	ITScore-PR	DARS-RNP	3dRPC-Score	
10	41.67	41.67	33.33	33.33	37.50
20	50.00	41.67	33.33	41.67	41.67
30	58.33	41.67	50.00	41.67	47.92
40	58.33	41.67	58.33	41.67	50.00
50	58.33	41.67	58.33	41.67	50.00

Supplementary Table S10. The PDB IDs of the unbound cases with relatively large interface interactions in RNA-protein complex benchmark I for the performance of DRPScore, ITScore-PR, DARS-RNP, and 3dRPC-Score.

1T0K, 3LRR, 1YVP, 1DFU, 1RC7, 1G1X, 1R9F, 2AZ0, 3CIY, 2RFK, 2GJW, 3LWR
